# Soil microbiota plays a key regulatory role in the outbreak of tobacco root rot

**DOI:** 10.3389/fmicb.2023.1214167

**Published:** 2023-09-15

**Authors:** Pengfei Li, Songsong Gu, Yanmei Zhu, Tianyang Xu, Yishuai Yang, Zhengqiang Wang, Xiangdong Deng, Bin Wang, Wei Li, Wenqiang Mei, Qiulong Hu

**Affiliations:** ^1^Wenshan Tobacco Company of Yunnan Province, Wenshan, Yunnan, China; ^2^Key Laboratory for Environmental Biotechnology, Research Center for Eco-Environmental Sciences, Chinese Academy of Sciences, Beijing, China; ^3^College of Horticulture and Landscape Architecture, Hunan Agricultural University, Changsha, Hunan, China

**Keywords:** tobacco, prokaryotes, Fungi, protist, network, root rot

## Abstract

**Introduction:**

Root rot caused by the fungal pathogen *Fusarium* sp. poses significant challenges to tobacco cultivation in China, leading to major economic setbacks. The interplay between this pathogen and the wider soil microbial community remains poorly understood.

**Methods:**

High-throughput sequencing technology was utilized to evaluate soil prokaryotic, fungal, and protistan communities. We compared microbial communities in infected soils to those in healthy soils from the same field. Additionally, the influence of pH on the microbial communities was assessed.

**Results:**

Infected soils displayed elevated levels of soil nutrients but diminished observed richness across prokaryotic, fungal, and protistan groups. The pathogenic fungi *Fusarium solani f* sp. *eumartii’s* abundance was notably increased in infected soils. Infection with *F. solani* significantly altered the soil’s microbial community structure and interactions, manifested as a decrease in network scale and the number of keystone species. An evaluation of prokaryotes’ role in *F. solani’s *invasion revealed an increased number of connecting nodes in infected soils. Additionally, relationships between predatory protists and fungi were augmented, whereas predation on *F. solani* declined.

**Discussion:**

The study underscores the significance of comprehending the interactions among soil microorganisms and brings to light the susceptibility of soil microbial communities to pathogen invasion. It offers insights into the multifaceted relationships and potential vulnerabilities within the soil ecosystem in the context of *Fusarium* sp. invasion.

## 1. Introduction

Tobacco (*Nicotiana tabacum* L.) is one of the most widely cultivated and economically important cash crops worldwide (Tong et al., 2016; Liu P. et al., 2020). However, tobacco root rot disease, caused by soilborne fungal pathogens such as *Fusarium solani f* sp. *eumartii* and *Phytophthora nicotianae*, has become a major threat to the tobacco industry, leading to significant yield losses (3–5%) and economic damage (Romberg and Davis, 2007; D’Ippólito et al., 2010). Root rot disease can cause wilting, stunting, and death of tobacco plants, with the infected roots turning brown or black and then rotting, ultimately leading to plant death (Bodah, 2017). Research has been conducted to understand the causal agents, epidemiology, and management strategies for tobacco root rot disease. Several studies have identified *Fusarium* species as the primary causal agent of root rot disease in tobacco plants, while others have reported the involvement of *Phytophthora* and *Rhizoctonia* species (Tan et al., 2021, 2022). The incidence and severity of disease are affected by a variety of factors, including soil pH, moisture, temperature, and the presence of other soilborne pathogens (Burdon and Zhan, 2020; Pokhrel, 2021). Additionally, recent research, such as the findings reported in ‘Legume Root Rot Control Through Soil Management for Sustainable Agriculture’ (Naseri, 2019), highlights the interaction of *F. solani* with agro-ecological factors on a larger scale.

In the context of addressing this challenge, understanding the interactions between various nutritional factors and soilborne pathogens is crucial. Nutrient availability, including nitrogen (N), phosphorus (P), potassium (K), and organic matter (OM), has been shown to profoundly affect soil microbial communities and subsequently influence plant health (Niu et al., 2017). To date, many studies have investigated the differences between healthy and diseased plants by examining bacterial and fungal communities (Hu et al., 2018; Tan et al., 2021; Gu et al., 2022). However, the current body of research lacks a comprehensive understanding of the regulatory mechanisms underlying environmental factors, such as the impact of different nutritional levels, particularly the effect of nutrient improvement, on disease suppression in the context of *F. solani* (Guo et al., 2022). Therefore, we intend to further explore and elucidate how soil nutrient levels, including N, P, K, and others, interact with *F. solani* populations both in the soil and within plant systems. Recognizing the significance of comprehending the interplay between nutrients and microbial communities for sustainable disease management in agriculture, our study distinguishes itself from prior research on *F. solani* and soil NPK interactions (Naseri and Hamadani, 2017). We aim to contribute novel insights by investigating these interactions in the specific context of our study system. Through this approach, we strive to elucidate the unique dynamics between nutrients and microbial communities, thereby providing valuable implications for the development of targeted strategies for disease suppression in agricultural systems.

Understanding the role of microbial communities in root rot disease is crucial for developing effective management strategies to reduce crop losses (Lareen et al., 2016; Li et al., 2021). Within the microbial community, bacteria, fungi, and protists play significant roles in the prevention and outbreak of root rot disease (Du et al., 2022). For example, changes in the relative abundance of specific taxa from bacterial and fungal communities have been shown to be associated with the healthy and diseased states of roots (Robbins et al., 2018; Liu X. et al., 2020). Many studies have also investigated the impact of microbial communities on root health, and some have shown promising results in reducing the incidence of root rot disease by manipulating microbial community composition (Lareen et al., 2016; Trivedi et al., 2020). For instance, the use of biocontrol agents, such as *Bacillus* spp. and *Trichoderma* spp., has been shown to promote plant growth and reduce root rot disease by suppressing the growth of pathogenic fungi (Fira et al., 2018; Kalantari et al., 2018). Protists, unicellular eukaryotic organisms, play pivotal roles in soil microbial dynamics (Bonkowski, 2004). Notably, some protists, such as those from the Cercozoa group, have emerged as key regulators of interactions between bacteria and fungi in the soil (Geisen et al., 2016). Their presence and activities could potentially influence the occurrence and severity of root rot diseases, emphasizing the need to delve deeper into their biocontrol potential against pathogens like *Fusarium* root rot (Deveau et al., 2018; Guo et al., 2022). Furthermore, recent studies have emphasized the critical role of environmental factors in determining the behavior and pathogenicity of *F. solani* (Gamboa-Becerra et al., 2021). Specifically, soil pH, organic matter, temperature, and moisture have been found to significantly influence the interactions of *F. solani* with microbial communities and plants (Yan and Nelson, 2020; Wang et al., 2023). The composition and structure of microbial communities can be altered by changes in these factors, which in turn can impact the incidence of root rot disease. In summary, the roles of bacteria, fungi, and protists in root rot disease prevention and outbreak are critical and interdependent. However, despite significant progress in understanding the role of microorganisms in root rot disease, much remains unknown about the specific mechanisms involved.

Network analysis methods have become increasingly popular in ecological studies, as they provide a useful framework for understanding the complex relationships between the different organisms in a community (Deng et al., 2016). Random matrix theory (RMT) has been applied in network analysis to examine the structure of random networks and compare them to real-world ecological networks (Deng et al., 2012). Meanwhile, the identification and evaluation of dual networks (IDEN) has been used to study bipartite networks in ecological systems, which involve two different biological domains, such as bacteria, fungi, and protists (Feng et al., 2022). In addition to their application in ecological research, network analysis methods have also been used to study the stability and complexity of ecological communities (Herren and McMahon, 2017; Yuan et al., 2021). Robustness and cohesion are two important aspects of community stability and complexity, respectively. Robustness refers to the ability of a community to maintain its structure and function in the face of perturbations, such as disturbances or species loss (Mumby et al., 2014; Yan et al., 2022). Cohesion, on the other hand, refers to the degree of interconnectedness between species within a community, and is a measure of how tightly integrated the community is (Hernandez et al., 2021). Network analysis methods such as RMT random networks and IDEN bipartite networks can be used to quantify these aspects of community stability and complexity (Deng et al., 2012; Feng et al., 2022). By using these methods, researchers can gain insights into the factors that contribute to the robustness and cohesion of ecological communities, and identify the potential strategies that maintain their stability and complexity in the face of environmental change (Yuan et al., 2021). Overall, network analysis methods have become an increasingly important tool for studying ecological systems (Xing and Fayle, 2021). By characterizing the complex patterns of interactions between species in these systems, network analysis can provide insights into the structure and function of ecological communities, as well as the processes that govern their dynamics (Tobias et al., 2014; Wang, 2018). Furthermore, network analysis methods can be used to investigate the stability and complexity of ecological communities, providing valuable information for conservation and management efforts (Yuan et al., 2021). As such, network analysis methods have the potential to contribute significantly to our understanding of ecological systems and inform efforts to protect and manage them.

The objective of this study was to shed light on the characteristics of prokaryotic, fungal, and protistan communities in the soil of healthy tobacco plants and those infected by *F. solani.* Specifically, we aimed to achieve the following: (i) use molecular ecological network analysis to reveal the associations among species of prokaryotic, fungal, and protistan communities; (ii) explore the associations between fungi and other microbial groups, including prokaryotes and protists, through interdomain ecological network (IDEN) analysis; (iii) study the associations between the pathogen *F. solani* and prokaryotes and predatory protists through sub-network analyses; and (iv) investigate how environmental factors regulate these mutual relationships. Through the accomplishment of these objectives, we aim to offer a novel strategy and theoretical foundation for enriching the investigation of microbial resources in tobacco soil. Additionally, we aim to explore antagonistic microbial resources that specifically target *F. solani* within the context of our well-defined pathosystem.

## 2. Materials and methods

### 2.1. Site characterization

The study was conducted in the Wenshan Zhuang and Miao Autonomous Prefecture of Yunnan-Kweichow Plateau (23°15′ N, 104°35′ E) in Yunnan province, China. The site has a subtropical climate with an elevation of 1,186 m, an average temperature of 19°C, a frost-free period of 356 days, sunshine of 2228.9 h, and an average annual rainfall of approximately 779 mm. The dominant soil type in the area is Calcareous soil, as classified by FAO (Food and Agriculture Organization of the United Nations).

### 2.2. Sample collection

In late July 2021, the study was carried out in a total of 8 tobacco growing plots where underwent multiple cropping pattern (tobacco and wheat) and rotational cropping system (tobacco and maize). All experimental plots had received similar agronomic management. Prior to the main study, pre-tests were conducted to assess the symptom of root rot in the tobacco fields, confirming the almost the same level of tobacco root rot infection. Soil samples were then collected from both healthy and infected tobacco soils using a random sampling strategy. A soil auger with a 5 cm inner diameter and able to collect soil to a 20 cm depth was used at each point to collect tobacco soil. Multiple soil samples (n = 5) were randomly collected from different points within each plot, spanning the 0–20 cm soil layer. These samples were then combined to create a composite sample representative of each plot. In total, 17 composite soil samples (8 healthy and 9 infected soil samples) were collected. The collected samples were divided into two parts. One part was processed immediately to measure the physicochemical characteristics of the soil, while the other part was stored at −80°C for subsequent DNA extraction to ensure preservation of the genetic material and to allow for further analysis.

### 2.3. Soil physicochemical analyses

To determine the soil physicochemical properties, various parameters including pH, soil total organic carbon (TOC), soil organic matter (OM), total nitrogen (TN), total phosphorus (TP), total potassium (TK), nitrate nitrogen (NO_3_^−^-N), ammonia nitrogen (NH_4_^+^-N), available phosphorus (AP), and available potassium (AK) were measured. The methods previously described by Du et al. (2022) were employed for this purpose. The quantification of all soil properties was conducted at the Institute of Soil Science, Chinese Academy of Sciences located in Nanjing, China. This ensured accuracy and consistency in the results obtained.

### 2.4. DNA extraction, PCR amplification, and sequencing

To extract soil total DNA, 0.5 g of mixed soil was used with the Mobio DNeasy® PowerSoil® Kit. The 16S rRNA genes of prokaryotes (515F: 5’-GTGCCAGCMGCCGCGGTAA-3′, 806R: 5’-GGACTACHVGGGTWTCTAAT-3′), ITS genes of fungi (5.8F, 5’-AACTTTYRRCAAYGGATCWCT-3′, 4R: 5’-AGCCTCCGCTTATTGATATGCTTAART-3′), and 18S genes of protistan (first step primers: 615F: 5’-AGTGTCGATTCGGTTAAAARGCTCGTAGTYG-3′, 963R: 5′-:AAGATCGTACTGAAGARGAYATCCTTGGTG-3′; second step primers: 615F: 5’-AGTGTCGATTCGGTTAAAARGCTCGTAGTYG-3′, 947R: 5’-AAGARGAYATCCTTGGTG-3′) were amplified using universal primers, which were supplemented with sample-specific barcodes (Zhou et al., 2021). The samples were sent to Magigene Biotechnology Co., Ltd. (Guangzhou, China) for sequencing on the Illumina Hiseq platform. This ensured high-quality sequencing, and the use of sample-specific barcodes allowed for identification and analysis of each sample individually.

### 2.5. Sequence processing

In total, 51 samples (17 samples × 3 microbial groups) were obtained. All the raw reads of 16S rRNA, ITS, and 18S genes were uploaded to a publicly available sequence analysis pipeline^1^ integrated with various bioinformatics tools (Feng et al., 2017). Firstly, the reads were assigned to samples according to their barcodes using the tool “Detected barcodes,” after which the barcode sequences were removed. Second, forward and reverse reads of the same sequence were combined using the FLASH tool (Magoč and Salzberg, 2011), and the combined reads without ambiguous bases were filtered by using the Btrim tool (Kong, 2011). Then, the Greengene database (DeSantis et al., 2006), ITS RefSeq database (Schoch et al., 2014), and PR2 database (Guillou et al., 2012) were used as references for chimera checking for the prokaryotic, fungal, and protistan communities, respectively. Singletons were retained as rare species (Jousset et al., 2017), and the clustering of sequences into operational taxonomic units (OTUs) was performed using UPARSE (Edgar, 2013) at a 97% threshold. Moreover, for ITS gene sequences, the ITSx tool was used to identify ITS sequences and extract the ITS regions. After obtaining OTU tables, we randomly resampled the reads to 50,000, 12,200, and 50,000 sequences for per prokaryotic, fungal, and protistan datasets, respectively. The Ribosomal Database Project (RDP) classifier was used to assign prokaryotic, fungal, and protistan OTUs with the Greengene ribosomal database (Wang et al., 2007), UNITE database (Abarenkov et al., 2010) and PR2 database (Guillou et al., 2012), respectively, with confidence values >0.8.

### 2.6. Molecular ecological network and inter-domain ecological network construction

To construct molecular ecology networks, we used the random matrix theory (RMT) approach described by Deng et al. (2012) for constructing intra-domain ecological networks (MEN) and the SparCC method for compositional data described by Friedman & Alm (Freilich et al., 2018) for constructing inter-domain ecological networks (IDENs). The open-access analysis pipeline (See Footnote 1) (Feng et al., 2022) was used for network construction. We filtered the Spearman correlations of the RMT results for the microbial communities using threshold values of *r* > = 0.95, 0.89, and 0.89 for the prokaryotic, fungal and protistan communities, respectively. The SparCC results of prokaryotic-fungal community and protistan-fungal community were filtered by the threshold values of *r* > = 0.8 and 0.7, respectively, and a false discovery rate < 0.05. We visualized the resulting networks using Gephi (0.9.2) and Cytoscape (3.5.1). To assess the significance of each observed MEN and IDEN, we used the Maslov-Sneppen approach to produce 100 randomly rewired networks (Bascompte et al., 2003), and each index was examined for the topological properties of each of the 100 random IDENs. We classified nodes into four subcategories: peripherals, connectors, module hubs, and network hubs, based on their values for within-module connectivity (Zi) and among-module connectivity (Pi) (Olesen et al., 2007). Module hubs, connectors, and network hubs were considered as keystone species in molecular ecological networks. We also used cohesion, a method to measure the degree of cooperative behaviors or competitive interactions correlation (Herren and McMahon, 2017), which could indirectly represent complexity.

To evaluate community resistance, we used robustness indices as described by previous studies (Dunne et al., 2002; Deng et al., 2012). The robustness index was calculated in two steps. First, we computed the abundance-weighted mean interaction strength of each node i (wMISi) using the relative abundance of species j (bj) and the association strength between species i and j (sij), as measured by Pearson correlation coefficient.


(1) $$wMIS_{i}=\frac{\sum_{j\neq i}b_{j}s_{ij}}{\sum_{j\neq i}b_{j}}$$

Second, we removed nodes with wMISi values ≤0 from the network, and reported the proportion of the remaining nodes as the network’s robustness.

Additionally, we used the cohesion index to quantify network complexity, which is a null model-corrected metric that considers abundance-weighting (Herren and McMahon, 2017). This index allowed us to assess the degree of connectivity among community members. We calculated two cohesion values (positive and negative) based on pairwise correlations. Positive correlations can indicate facilitation/mutualism among taxa reflecting ecological or functional similarity (Barberán et al., 2012; Durán et al., 2018), while negative correlations can suggest competition reflecting divergent niche requirements among taxa (Zelezniak et al., 2015; Freilich et al., 2018). We calculated the two cohesion values using the following equations:


(2) $$c_{j}^{pos}=\overset{n}{\underset{i=1}{\sum }}a_{i}\cdot {\bar{r}}_{i,r}>0\;\left(\mathrm{Positive}\;\mathrm{Cohesion}\right)$$

and


(3) $$c_{j}^{neg}=\overset{n}{\underset{i=1}{\sum }}a_{i}\cdot {\bar{r}}_{i,r}<0\;\left(\mathrm{Negative}\;\mathrm{Cohesion}\right)$$

Where *a_i_* is the abundance of OTU *i* in the sample j and ${\bar{r}}_{i,r}$ is the connectedness.

### 2.7. Statistical analysis

An open-access analytical pipeline (See Footnote 1) (Feng et al., 2017) was used for all statistical analyses. Alpha-diversity of the microbial communities was assessed by computing the Richness index, which counted the observed species in the resampled OTU table. To examine the correlation between soil characteristics and microbial communities, Mantel test was performed. Community dissimilarity was evaluated using MRPP, ANOSIM, and PERMANOVA (Anderson, 2001). ANOVA was employed to test for differences in soil physicochemical variables and diversities between each pair of treatments, and Tukey post-hoc tests and LSD test were used to determine significant differences. The one-sample Student’s *t*-test was used to determine the features of each treatment pair, and the difference between observed and random networks. The Random-Forests (RF) algorithm was utilized to identify the most significant environmental factors influencing the abundance of pathogenic fungi (Statnikov et al., 2008).

## 3. Results

### 3.1. Soil heterogeneity found in physicochemical properties of healthy and infected plants from the same field

Upon comparing the soil physicochemical properties of healthy and diseased plants collected from the same field, it was found that the infected soil had significantly higher levels (ANOVA, *p* < 0.05) of OM, TOC, TN, TP, TK, AP, AK, NO_3_^−^-N, and NH_4_^+^-N than the healthy soil, while there was no significant difference in pH (Supplementary Figure S1). This indicated the presence of significant heterogeneity in soil texture and nutrient content within the field.

### 3.2. Microbial communities’ difference between healthy and infected tobacco soil

Using a 97% similarity threshold, we obtained 10,576 prokaryotic OTUs, 1,272 fungal OTUs, and 5,406 protistan OTUs across the 51 samples (17 soil samples × three microbial communities). The observed richness of prokaryotic, fungal and protistan groups was higher in heathy soil than infected soil (Supplementary Figure S2, ANOVA, *p* > 0.005). Among the three microbial groups, prokaryotes exhibited the highest observed richness of species, followed by protists, while fungi showed the lowest diversity (Supplementary Figure S2). We used NMDS (non-metric multidimensional scaling) based on Bray–Curtis dissimilarity to visualize the differences in group structure. The results showed significant differences in community structure between healthy and infected soil prokaryotes (*stress = 0.057*), fungi (*stress = 0.174*), and protists (*stress = 0.062*) (Supplementary Figure S3). Further analysis using a dissimilarity test revealed significant differences between healthy and infected soil (Supplementary Table S1).

Taxonomic analysis was conducted at the phylum level to determine the relative abundances of taxa in all three microbial groups. In tobacco soil, the dominant (relative sequence abundance ≥1%) prokaryotic phyla were Proteobacteria (H: 43.10%, I: 45.32%), Acidobacteria (H: 10.86%, I: 14.78%), Actinobacteria (H: 13.94%, I: 9.69%), Gemmatimonadetes (H: 7.55%, I: 6.40%), Chloroflexi (H: 6.67%, I: 6.06%), Unclassified (H: 6.11%, I: 5.46%), Bacteroidetes (H: 4.48%, I: 4.46%), Saccharibacteria (H: 2.07%, I: 2.70%), Nitrospirae (H: 1.77%, I: 1.70%), Firmicutes (H: 1.23%, I: 0.98%), and Verrucomicrobia (H: 1.19%, I: 0.98%) (Supplementary Figure S4A). The dominant fungal phyla were Ascomycota (H: 56.67%, I: 54.93%), Unclassified (H: 19.18%, I: 30.52%), Basidiomycota (H: 5.40%, I: 12.05%), Zygomycota (H: 13.63%, I: 2.03%), and Chytridiomycota (H: 5.10%, I: 0.46%) (Supplementary Figure S4B). The dominant protistan phyla were Streptophyta (H: 75.49%, I: 56.45%), Metazoa (H: 5.07%, I: 15.62%), Cercozoa (H: 5.47%, I: 11.35%), Ascomycota (H: 4.04%, I: 7.18%), Ochrophyta (H: 1.47%, I: 2.69%), Ciliophora (H: 1.83%, I: 1.90%), Lobosa (H: 1.93%, I: 1.36%), Chlorophyta (H: 0.49%, I: 2.76%), Pseudofungi (H: 1.01%, I: 0.47%), and Unclassified (H: 1.50%, I: 1.53%) (Supplementary Figure S4C). To investigate the impact of pathogenic fungi, we focused on the fungal community and identified species with relative abundance ≥1% (Supplementary Figure S5A). Notably, the pathogenic fungus *Fusarium solani f* sp. *eumartii* was present in both healthy and infected soils, but its relative abundance, at 8.67%, in infected soil was significantly higher (ANOVA, *p* < 0.001) compared to healthy soil (0.42%) (Supplementary Figure S5B). In summary, the presence of pathogenic fungi reshaped the prokaryotic, fungal, and protistan communities in tobacco soil.

### 3.3. The impact of environmental factors on microbial community

**Figure 1:**
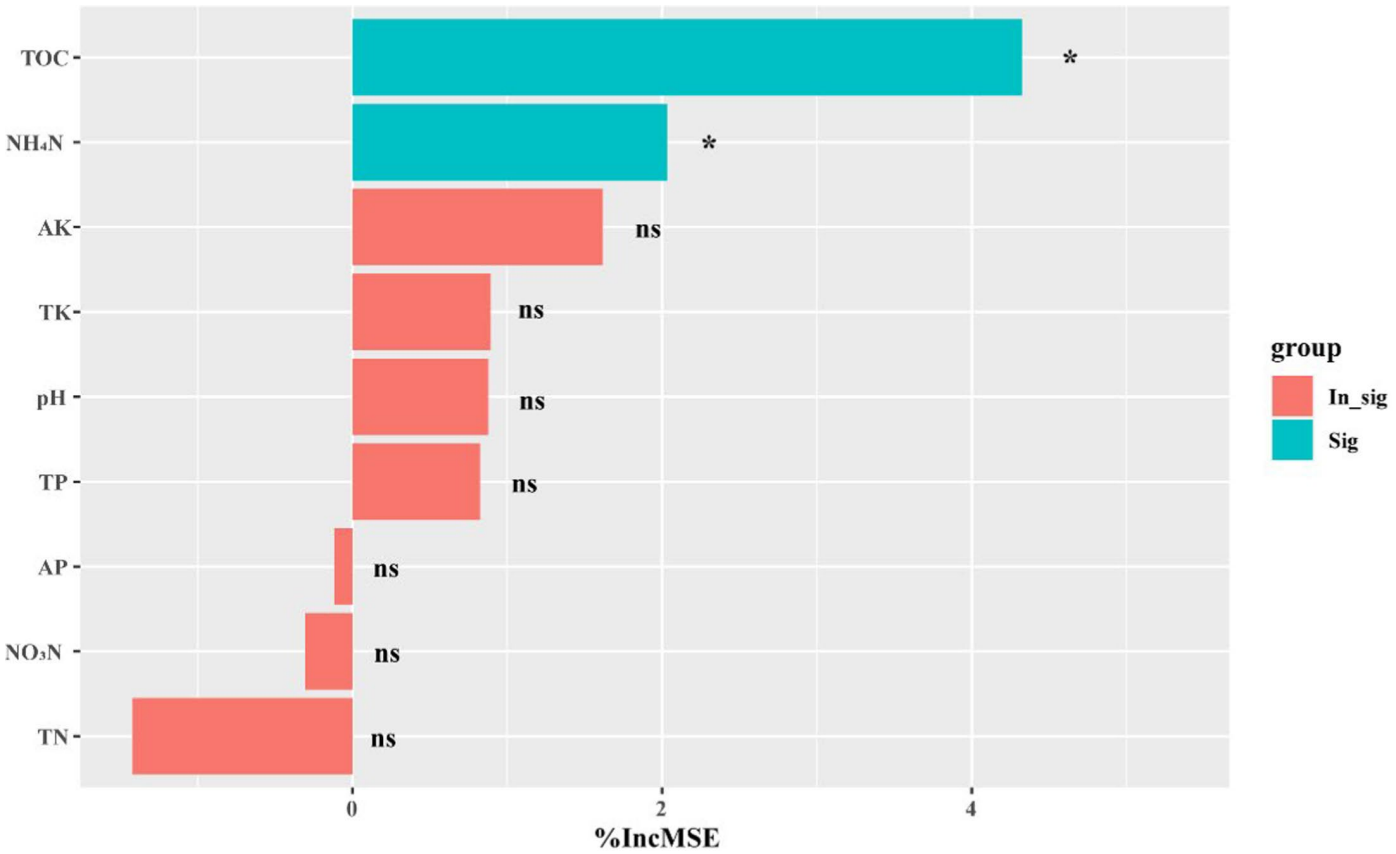
Random forest module results. %IncMSE represents the average percentage increase in the model’s error after using each feature to perform a split. The blue color represents the significant correlation between the environmental factor with the relative abundance of *Fusarium solani f* sp. *eumartii*. The bar on the left represents the negative correlation, while the bar on the right represents the positive correlation. The levels of significance are indicated as 0.001***, 0.01**, and 0.05*.

To assess the impact of environmental factors on soil microbial community composition and structure, we employed the Mantel test using Bray–Curtis dissimilarity (Supplementary Table S2). Our results demonstrate that pH exerted a significant influence on the prokaryotic (*r* = 0.2667, *p* = 0.005), fungal (*r* = 0.2984, *p* = 0.002), and protistan (*r* = 0.1438, *p* = 0.034) communities in both healthy and infected soils. Notably, we also observed that the influence of TOC, NO_3_^−^-N, and NH_4_^+^-N on the microbial community varied according to the specific microbial group under consideration. For instance, TOC and NH_4_^+^-N exhibited a stronger effect on the fungal community (*r* = 0.2382, *p* = 0.033; *r* = 0.1874, *p* = 0.029) than on the prokaryotic community (*r* = −0.0291, *p* = 0.525; *r* = 0.1111, *p* = 0.128) or protistan community (*r* = −0.2329, *p* = 0.969; *r* = −0.0858, *p* = 0.776). NO_3_^−^-N exerted a stronger effect on the fungal (*r* = 0.1837, *p* = 0.018) and protistan (*r* = 0.1538, *p* = 0.048) communities than on the prokaryotic community (*r* = 0.2382, *p* = 0.128). Furthermore, we observed that TOC and NH_4_^+^-N were strongly associated with shifts in the relative abundances of pathogenic fungi (i.e., *F. solani*) (Figure 1). Collectively, our findings underscore the intricate interplay between environmental factors and microbial communities in tobacco soils, and suggested that pH, TOC, NO_3_^−^-N, and NH_4_^+^-N may be pivotal in shaping the microbiome of this ecosystem.

Variance partitioning analysis (VPA) was conducted to investigate the relative impact of environmental factors on prokaryotic, fungal, and protistan communities in tobacco soils. The results showed that only a portion of the variation within the communities (54.97% for prokaryotes, 56.89% for fungi, and 49.15% for protists) could be explained by environmental factors (Supplementary Figure S6). Our analysis of environmental stress (pH) and soil nutrient (TOC, TN, TP, TK, AP, AK, NO_3_^−^-N, and NH_4_^+^ -N) models revealed that pure environmental stress accounted for a mean of 9.40, 6.58, and 7.98% of the variation in prokaryotic, fungal, and protistan communities, respectively, while pure soil nutrients explained a mean of 42.67, 49.06, and 40.88% of the variation. These results suggest that a large proportion of the variance (45.03, 43.11, and 50.85% for the prokaryotic, fungal, and protistan communities, respectively) remained unexplained, indicating the potential importance of neutral or stochastic processes in community aggregation.

### 3.4. Impact of *Fusarium solani* infection on soil microbial community interactions and stability

We employed molecular ecological networks (MENs) to study the changes in the interactions between microbial communities following infection by pathogenic fungi. To ensure comparability of different networks, we established MENs for both healthy and infected soil samples using the same threshold values (0.95 for prokaryotic, 0.89 for fungal, and 0.89 for protistan communities). The overall topological indices revealed that the average path lengths (GD) of all networks were between 4.935 and 12.582. These values were close to the logarithms of the total number of network nodes and were higher than those of their corresponding random networks (Supplementary Table S3). This indicated that the MENs showed the typical properties of small-world networks. The modularity of all networks for prokaryotic, fungal, and protistan communities ranged from 0.681 to 0.901, which was significantly higher than the modularity value of their corresponding randomized networks. This suggests that all constructed networks had modular topology. These key topological properties allowed us to conduct further analysis on the constructed networks.

**Figure 2:**
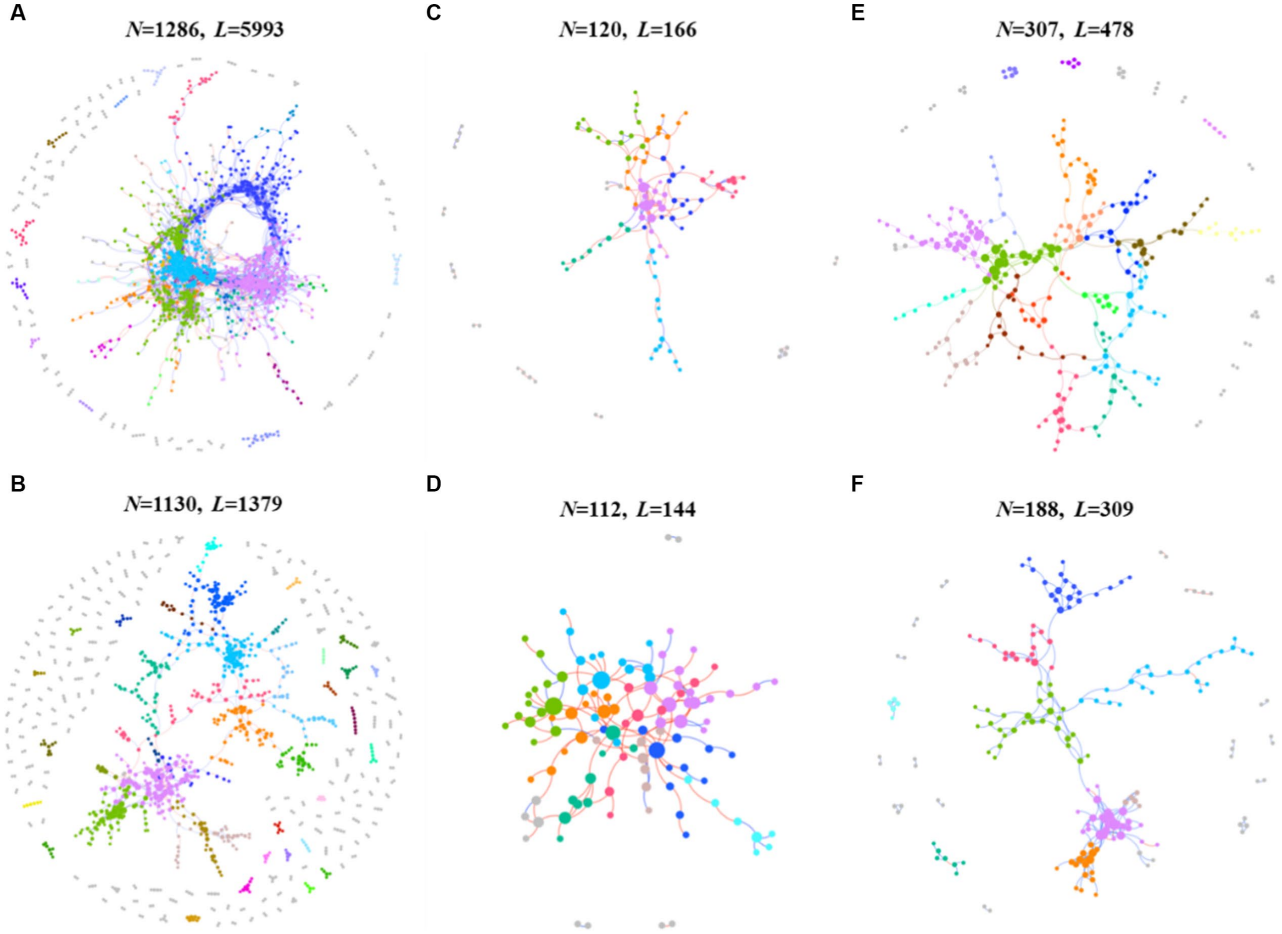
The intra-domain network analysis of prokaryotic, fungal and protistan communities. **(A)** The prokaryotic network of healthy samples. **(B)** The prokaryotic network of infected samples. **(C)** The fungal network of healthy samples. **(D)** The fungal network of infected samples. **(E)** The protistan network of healthy samples. **(F)** The protistan network of infected samples. The different colors represent different modules, while modules with five nodes or less are grey. Node size indicates the node degree of node. N, node; L, links.

**Figure 3:**
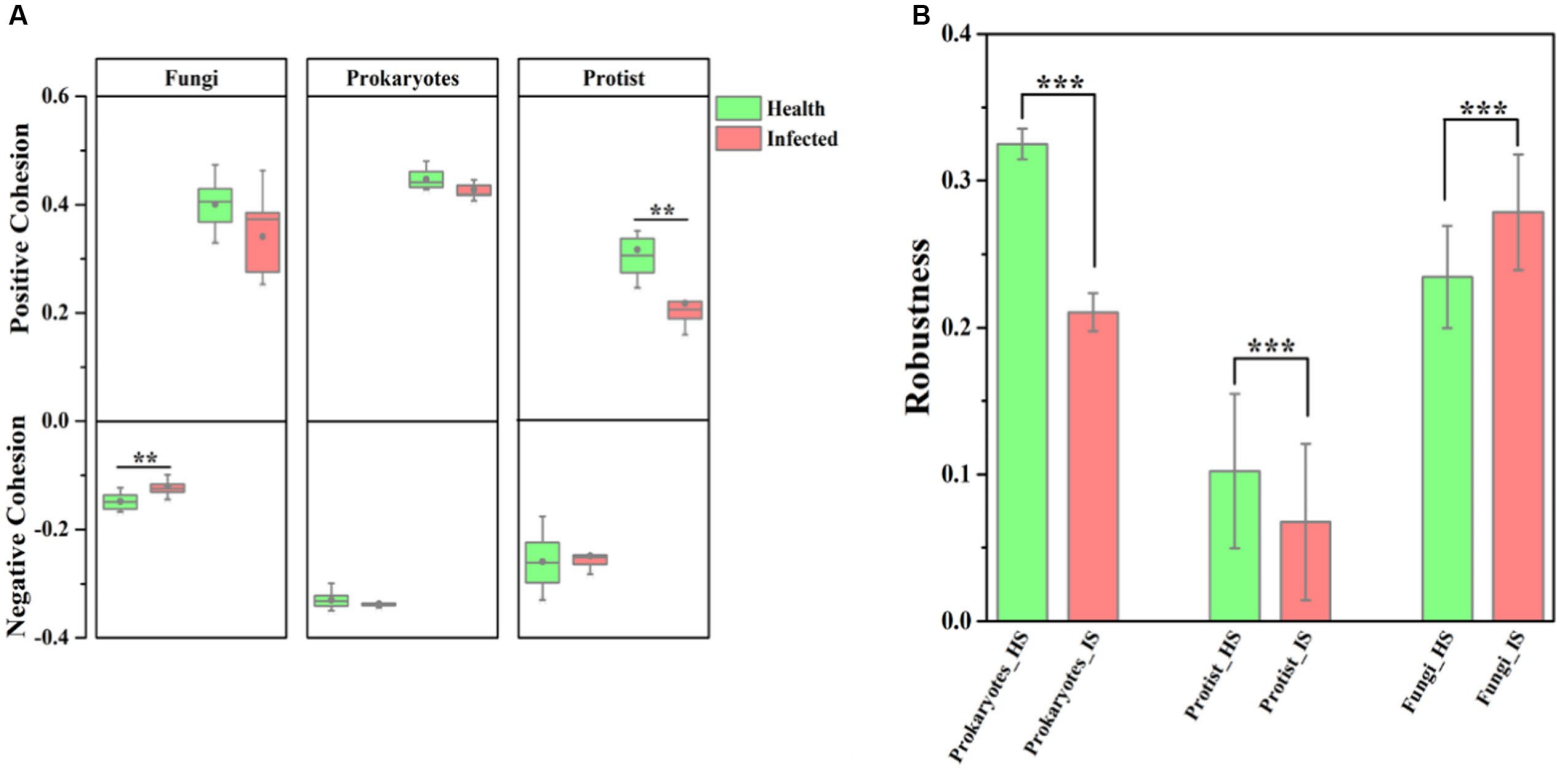
The complexity and stability of soil microbial community. **(A)** Cohesion analysis of the microbial communities, where green represents healthy samples and the red represents infected samples. **(B)** Robustness analysis of the microbial communities. HS, healthy soil; IS, infected soil. The levels of significance are indicated as 0.001***, 0.01**, and 0.05*.

The application of network analysis revealed a reduction in the scale of the network (i.e., number of nodes and edges) following *F. solani* infection (Figure 2). The *Z_i_* - *P_i_* analysis results demonstrated that the number of keystone species in healthy soil was higher for prokaryotic and protistan community networks (29 and 4 nodes, respectively) compared to infected soil (8 and 1 nodes, respectively). However, for fungal community networks, the number of keystone species was higher in infected soil (10 nodes) than healthy soil (5 nodes) (Supplementary Table S4). Cohesion analysis revealed a higher absolute value of negative cohesion for fungal communities in healthy soil, indicating increased competition among fungi compared to infected soil. Conversely, healthy soil exhibited a higher absolute value of positive cohesion for protistan communities, indicating increased cooperation among protists compared to infected soil (Figure 3A). The ANOVA results indicated that the robustness of prokaryotic and fungal communities was significantly higher in healthy soil than in infected soil (*p* < 0.05), indicating greater community stability in healthy soil. However, in protistan communities, robustness was lower in healthy soil (Figure 3B). These findings suggest that infection by *F. solani* has a significant impact on microbial community structure and interactions in the soil, and highlighted the vulnerability of the microbial communities to pathogen invasion.

The results of network analysis indicated that in healthy and infected soils, the potential pathogen *F. solani* was negatively correlated with several fungal species, including *Monacrosporium thaumasium*, *Rhizophlyctis rosea isolate*, *Nectriaceae* sp., *Arrhenia* sp., *Talaromyces assiutensis isolate*, and *Leptodophora orchidicola*. On the other hand, *F. solani* was positively correlated with *Scutellinia nigrohirtula* and three unclassified fungal genera (Supplementary Figures S7A,B). These positive and negative correlations suggested that certain fungi may play important roles in either assisting or inhibiting fungal root rot infections. We suggest that pathogen invasion may be aided by native microbial members that are positively correlated with *F. solani*, potentially through assistance in colonization or mutualistic relationships that are enriched during the infection process.

### 3.5. The role of prokaryotes in the invasion of *Fusarium solani*

**Figure 4:**
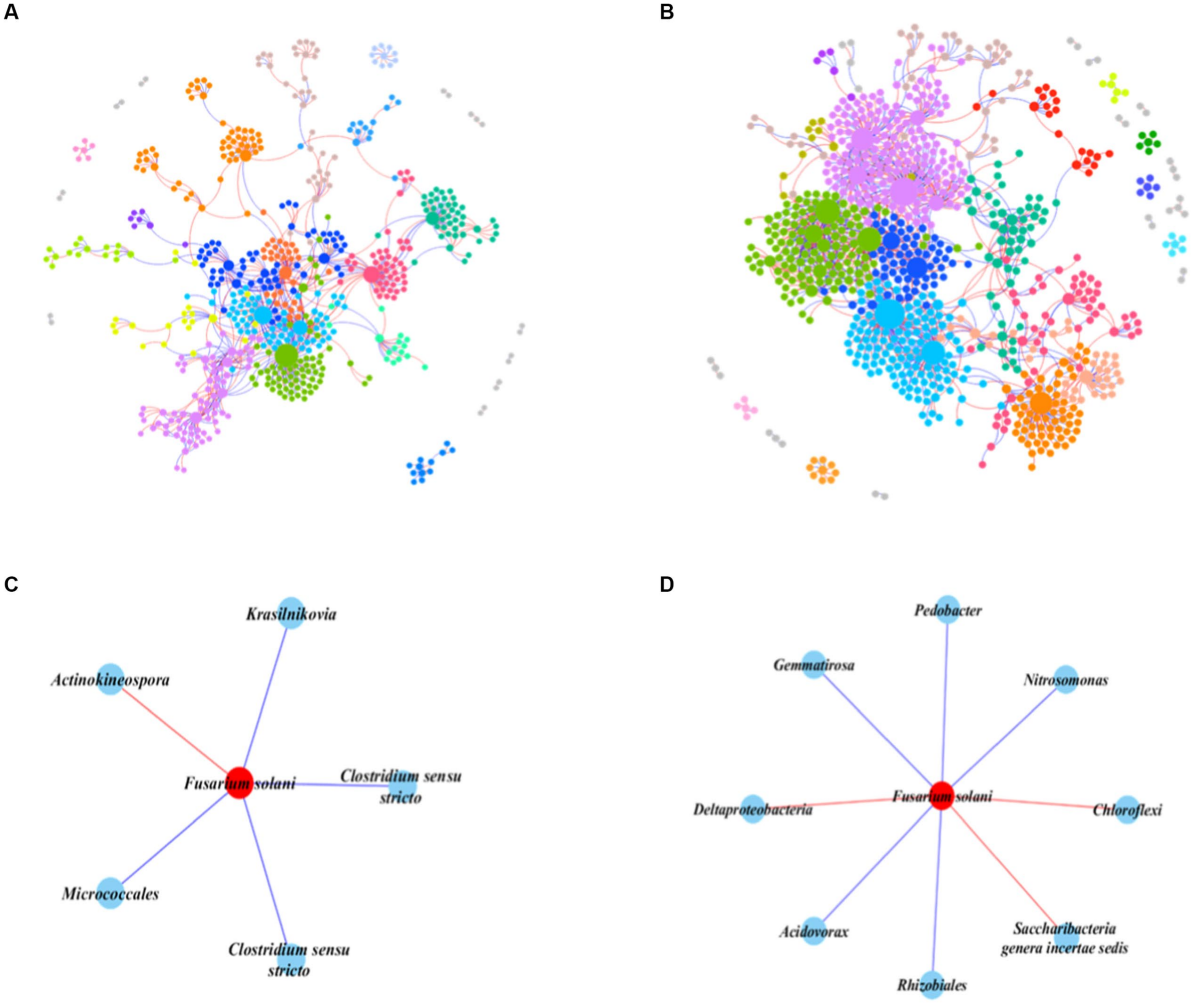
Interdomain ecological networks of the prokaryotic-fungal associations of healthy and infected samples. **(A)** The whole network of healthy samples. **(B)** The whole network of infected samples. **(C)** The *F. solani*-prokaryotes sub-network of healthy samples. **(D)** The *F. solani-prokaryotes* sub-network of infected samples. The different color represents the different modules, while modules with five nodes or less are grey. Node size indicates the node degree of node. Red links in the sun-networks indicate negative correlations.

We constructed an IDEN of the prokaryotic-fungal community to evaluate the importance of prokaryotes in the invasion of *F. solani*. The networks in healthy and infected samples both exhibited basic bipartite topological structures (Supplementary Table S5), but showed significant topological differences (Figures 4A,B). The healthy samples network had higher numbers of nodes and links (96 fungal nodes and 714 prokaryotic nodes, 1,273 links) than the infected network (72 fungal nodes and 522 prokaryotic nodes, 806 links), indicating more complex and tighter bacterial-fungal associations than in infected samples.

To gain deeper insights into the impact of prokaryotes on *F. solani*, we selected all nodes connected to *F. solani* and constructed a subnetwork (Figures 4C,D). The results showed that in the infected network *F. solani* possessed a greater number of connected nodes (8 nodes) than in the healthy network (5 nodes). Furthermore, one resistance bacterial species (based on the negative correlations) belonging to the genus *Actinokineospora* was found in healthy samples, while three resistance bacterial species (one unclassified species belonging to the class Deltaproteobacteria, one unclassified species belonging to the phylum Chloroflexi and one species belonging to the genus *Saccharibacteria*). There were four prokaryotic species (one species belonging to the genus Clostridium, two species belonging to the genus *Krasilnikovia* and one species belonging to the class Actinobacteria) positively connected with *F. solani* in the healthy network, while five prokaryotic species (two species belonging to the genus *Gemmatirosa*, two species belonging to genus *Nitrosomonas* and one species belonging to genus *Acidovorax*) were positively connected with *F. solani* in the infected network. Overall, the subnetwork analysis revealed that in infected soils, *F. solani* had a higher number of connected nodes compared to healthy soils. Additionally, there were more resistance bacteria species positively connected to *F. solani* in infected soils than in healthy soils.

### 3.6. The role of predatory protists in the invasion of *Fusarium solani*

**Figure 5:**
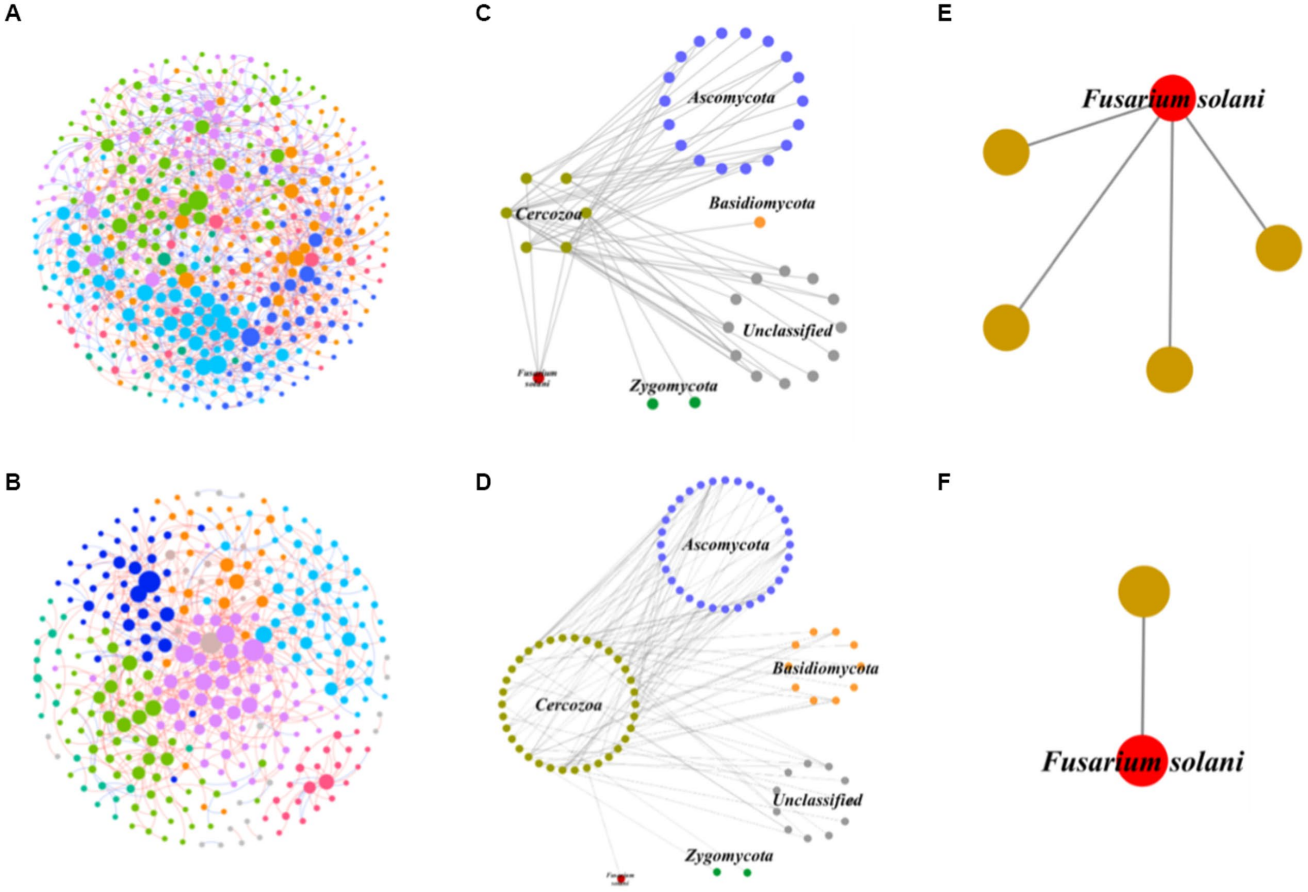
Interdomain ecological networks of the protistan-fungal associations of healthy and infected samples. **(A)** The whole network of healthy samples. **(B)** The whole network of infected samples. **(C)** The Cercozoa-fungi sub-network of healthy samples. **(D)** The Cercozoa-fungi sub-network of infected samples. **(E)** The sub-network of Cercozoa-pathogenic fungi (*F. solani*) of healthy samples. **(F)** The sub-network of Cercozoa-pathogenic fungi (*F. solani*) of infected samples.

In order to better understand the interactions between predatory protists and the fungal community, IDENs between predatory protists and the fungal community were constructed (Figures 5A,B). To do this, we selected 131 fungal and 344 protistan species from healthy samples, and 127 fungal and 188 protistan species from infected samples, to illustrate the associations between fungi and protists (Supplementary Table S5). To gain deeper insights into the impact of invasion on the predation relationship of predatory protozoa, we focused on nodes related to the Cercozoa and constructed a subnetwork (Figures 5C,D). In healthy soil, we found that 18 nodes of Ascomycota, 1 node of Basidiomycota, 12 nodes of Unclassified, 2 nodes of Zygomycota, and 1 node of *F. solani* were mainly associated with Cercozoa (6 nodes). However, in infected soil, we observed that 32 nodes of Ascomycota, 10 nodes of Basidiomycota, 11 nodes of Unclassified, 2 nodes of Zygomycota, and 1 node of *F. solani* were mainly associated with Cercozoa (34 nodes). These results suggested that the invasion of pathogenic fungi significantly increased the number of Cercozoa in soil, which in turn stimulated predation by predatory protists (Cercozoa) on the fungal community. Furthermore, the invasion seems to increase the predation of predatory protists (Cercozoa) on Ascomycota and Basidiomycota. Interestingly, the predation of *F. solani* was decreased in infected soil (Figures 5E,F), indicating that there was lower survival pressure on this pathogen. Overall, our findings shed light on the complex inter-domain interactions between predatory protists and the fungal community, and the impact of pathogenic fungi on these relationships.

### 3.7. Soil variables influence network connectivity in bipartite networks

We conducted partial Mantel tests (Table 1) to explore the associations between soil variables and the connectivity of all bipartite networks. Our analysis revealed that soil variables had a significant impact on the connectivity of the bipartite networks. However, the specific variables involved varied depending on the health status of the soil. In healthy prokaryotic-fungal networks, connectivity was significantly influenced by pH (*r* = 0.0544, *p* = 0.02), TK (*r* = 0.0611, *p* = 0.01), AK (*r* = 0.0529, *p* = 0.036), and NO_3_^−^-N (*r* = 0.081, *p* = 0.009). Conversely, the connectivity of infected prokaryotic-fungal networks was only influenced by NO_3_^−^-N (*r* = 0.0646, *p* = 0.044). For protistan-fungal networks, we found that connectivity was significantly influenced by TK (*r* = 0.063, *p* = 0.015), AP (*r* = 0.0577, *p* = 0.038), and AK (*r* = 0.0813, *p* = 0.006) in healthy soil. In contrast, in infected soil, connectivity was significantly influenced by TOC (*r* = 0.0619, *p* = 0.0394), AK (*r* = 0.057, *p* = 0.038), and NH_4_^+^-N (*r* = 0.0967, *p* = 0.009). Overall, these findings highlight the importance of considering soil health status when studying the associations between soil variables and the connectivity of bipartite networks. By identifying the specific soil variables that influence network connectivity, these results could inform soil management practices aimed at promoting a healthy and diverse soil ecosystem.

**Table 1:** The correlations between module eigenvalues and environmental traits in the healthy and infected samples networks.

Mantel	16S-ITS-HS		16S-ITS-IS		18S-ITS-HS		18S-ITS-IS	
	*r*	*P*	*r*	*P*	*r*	*P*	*r*	*P*
pH	0.0544	0.02*	0.0247	0.135	0.0526	0.053	−0.0582	0.979
TOC	−8.00E-04	0.429	−0.0336	0.908	−0.0407	0.96	0.0619	0.0394*
TN	−0.0165	0.734	0.0304	0.14	0.0121	0.312	−0.0544	0.947
TP	0.0048	0.38	0.0021	0.394	−0.0521	0.996	−0.0623	0.978
TK	0.0611	0.01	0.0077	0.315	0.063	0.015*	−0.0399	0.917
AP	0.0043	0.398	−0.0263	0.815	0.0577	0.038*	−0.0582	0.972
AK	0.0529	0.036*	0.0124	0.286	0.0813	0.006**	0.057	0.038*
NO_3_^−^-N	0.081	0.009**	0.0646	0.044*	−0.0013	0.476	−0.0399	0.876
NH_4_^+^-N	0.0142	0.274	0.0407	0.138	0.0339	0.116	0.0967	0.009**

16S-ITS-HS, the healthy inter-domain network of prokaryotic-fungal community; 16S-ITS-IS, he infected inter-domain network of prokaryotic-fungal community; 18S-ITS-HS, the healthy inter-domain network of protistan-fungal community; 18S-ITS-IS, he infected inter-domain network of protistan-fungal community; The levels of significance are indicated as 0.001***, 0.01**, and 0.05*.

## 4. Discussion

In the context of the existing literature on *Fusarium* solani root rot, this study sought to elucidate the intricate interactions between soil microbial communities and the incidence of this pathogenic infection in tobacco fields. Our objectives were guided by the need to address gaps in our understanding of how various soil factors influence the proliferation of *F. solani* and how microbial communities respond to the presence of this pathogen. By accomplishing these objectives, we aimed to provide a new strategy and theoretical support for enriching the study of tobacco soil microbial resources and for exploring antagonistic microbial resources targeting *F. solani*. The current research builds upon prior investigations that have examined the relationships between soil properties, microbial communities, and disease incidence. A careful review of the literature reveals a scarcity of research on the regulatory mechanisms of environmental factors such as different nutritional levels, particularly the impact of nutrient improvement on disease suppression (Guo et al., 2022). Additionally, while there are studies that have explored the impact of soil properties on the occurrence of *F. solani* (Tan et al., 2021, 2022), little attention has been given to the intricate network of interactions involving other microorganisms, such as bacteria, fungi, and protists, in the context of this pathogenic invasion.

Our findings have brought to light significant disparities in soil attributes between healthy and diseased plants within the same field. The soil hosting the infection exhibited notably higher levels of organic matter, total organic carbon, total nitrogen, total phosphorus, available potassium, available phosphorus, nitrate nitrogen, and ammonium nitrogen compared to the healthier soil. These pronounced differences underscore the existence of substantial heterogeneity in soil texture and nutrient content across the field, a phenomenon commonly referred to as “soil patchiness” (Eviner and Firestone, 2007; He et al., 2012). Soil patchiness can stem from various sources, including soil type, water distribution, chemical gradients, and topography (Laekemariam et al., 2017; Lefebvre et al., 2020). These variations can translate into discrepancies in plant growth (Wang et al., 2021; Cao et al., 2023). Importantly, soil patchiness plays a multifaceted role in shaping disease dynamics. In environments characterized by varied soil attributes, different regions of the same field may provide varying levels of vulnerability to pathogenic organisms (Tan et al., 2021). The localized enrichment of nutrients in certain patches might create more favorable conditions for the proliferation of pathogens such as *F. solani*. Conversely, patches with less favorable attributes may hinder pathogen growth. This dynamic interplay between soil patchiness and disease dynamics underscores the complexity of microbial interactions in the soil ecosystem and has important implications for disease management strategies.

The establishment of pathogenic *F. solani* in soil demonstrates significant repercussions on the soil’s microbial community (Tan et al., 2021). Our research underscores that the microbial community of afflicted plants contrasts starkly with that of their healthy counterparts, as evidenced by reduced microbial diversity and richness (Supplementary Figure S3 and Supplementary Table S1). This corroborates prior studies that indicate the presence of pathogenic fungi can curtail microbial diversity and reshape community structure (Mendes et al., 2018; Qu et al., 2020; Tan et al., 2022). This decline in diversity could emanate from the competitive edge that pathogenic fungi gain, possibly through the release of toxins or competition for resources (Bais et al., 2003; Fu et al., 2017). Furthermore, alterations in microbial community structure might stem from shifts in soil conditions induced by pathogenic intrusion, such as changes in pH or nutrient availability (Zhang et al., 2016). Our findings underscore that pathogenic fungal incursions exert a substantial influence on soil microbial communities, yielding diminished diversity and richness. These outcomes possess crucial implications for plant well-being and agricultural yield, considering the pivotal role of soil microbial communities in nutrient cycling, soil structure, and plant-microbe interplay. Further explorations are essential to unravel the mechanisms orchestrating these effects and to devise strategies for managing plant diseases borne in the soil.

This study casts light on the sway of environmental factors upon the composition and structure of microbial communities within tobacco soils (Wang et al., 2022). The results of our study demonstrate that soil pH, TOC, NO_3_^−^-N, and NH_4_^+^-N are key environmental factors that influence the composition and structure of microbial communities in tobacco soils. Our findings are consistent with previous studies that have identified soil pH as a major driver of microbial community structure (Christian et al., 2009; Fierer et al., 2013). Moreover, our results indicate that the influence of soil nutrients on microbial communities may vary depending on the specific microbial group under consideration (Zhou et al., 2021). For example, TOC and NH_4_^+^-N have a greater impact on fungal communities than on prokaryotic or protistan communities, while NO_3_^−^-N has a stronger effect on fungal and protistan communities than on prokaryotic communities. Our study also suggests that environmental factors account for a substantial, but incomplete, portion of the variation in microbial community structure (Chen and Gu, 2022). Variance partitioning analysis revealed that only a portion of the variation in microbial communities could be explained by environmental factors, indicating the potential importance of neutral or stochastic processes in community aggregation. These findings are consistent with previous studies that have suggested that neutral or stochastic processes may play a significant role in shaping microbial community structure (Nemergut et al., 2016; Zhou and Ning, 2017). Overall, our study highlights the intricate interplay between environmental factors and microbial communities in tobacco soils. Our findings suggest that soil pH, TOC, NO_3_^−^-N, and NH_4_^+^-N may be pivotal in shaping the microbiome of this ecosystem, and emphasize the need for further research to elucidate the underlying mechanisms driving these relationships.

This study also expounds on the transformative influence of pathogenic *F. solani* on the arrangement and dynamics of soil microbial communities (Tan et al., 2021, 2022). The contraction of network scale, indicated by diminished nodes and edges, subsequent to infection signifies a reduced complexity in microbial communities (Figure 2), a phenomenon echoed in earlier studies (Urich et al., 2008; Banerjee et al., 2018). Furthermore, the decline in keystone species numbers within prokaryotic and protistan community networks portends a diminution in their robustness and stability, given the crucial roles played by keystone species in sustaining ecosystem function and resilience (Deng et al., 2012; Banerjee et al., 2018). In contrast, the augmentation in keystone species numbers within fungal community networks could denote a response to pathogenic invasion, with these key species potentially stifling *F. solani* growth or facilitating the establishment of other beneficial fungal strains (Tan et al., 2022). Noteworthy examples, such as Monacrosporium thaumasium and Rhizophlyctis rosea isolate, could emerge as pivotal contributors in the microbial ecosystem of infected soils, offering novel strategies for curtailing fungal root rot outbreaks (Hu et al., 2020; Tan et al., 2022). Additionally, cohesion analysis unveils an intensified fungal competition in healthy soils, juxtaposed with heightened protist cooperation in infected soils (Figure 3A). This shift implies altered community interactions, manifesting as changes in microbial community composition and structure (Chapelle et al., 2016). Further insights arise from ANOVA results, pointing to the superior robustness of prokaryotic and fungal communities in healthy soils relative to infected ones (Figure 3B), potentially indicating the deleterious impact of *F. solani* on the stability of these microbial communities (Yan and Nelson, 2020; Saengchan et al., 2022). However, the augmented robustness of protistan communities in healthy soils may mirror their adeptness at withstanding pathogenic incursions and upholding stability (Guo et al., 2022).

The soil, a multifaceted ecosystem, houses a plethora of microorganisms, including bacteria and fungi, pivotal in nutrient cycling and plant prosperity (Effmert et al., 2012). Endophytic microorganisms further inhabit plant tissues, potentially conferring disease resistance or nutrient access (Bamisile et al., 2018). The interactions among fungi, bacteria, and protists within this milieu assume critical roles in microbial communication networks, integral to the equilibrium and functioning of these habitats (Deng et al., 2012). Our study spotlights the notable role of inter-domain interactions, interweaving prokaryotes, fungi, and protists, paramount to soil ecosystem dynamics. The encroachment of pathogenic fungi, exemplified by *F. solani*, disrupts these interactions, exerting transformative effects (Figures 4, 5). Investigation through the IDEN approach discerns intricate bacterial-fungal associations in healthy soils, while infection enriches *F. solani*’s connections with prokaryotic species (Figures 4C,D). This phenomenon suggests that pathogenic fungi might stimulate the growth of specific bacterial species, possibly fostering or suppressing fungal growth—a key insight into the regulation of *F. solani*’s expansion and virulence (Tan et al., 2021). Our discoveries corroborate earlier findings showcasing the vital role of prokaryotes in soil health by controlling fungal pathogens (Zahir et al., 2003; Niu et al., 2017). Notably, our study unveils that *F. solani* invasion escalates Cercozoa numbers, triggering predatory protists’ predation on fungal communities (Figures 5C,D). Remarkably, the predation of pathogenic *F. solani* declines in infected soil (Figures 5E,F), hinting at reduced survival pressure on the pathogen. These trends align with previous research underlining the influential role of predatory protists in steering soil microbial communities (Rønn et al., 2002; Geisen et al., 2015). Collectively, our study accentuates the indispensability of accounting for inter-domain interactions when scrutinizing soil microbial communities, particularly under the impact of pathogenic fungi.

Several studies have shown that soil physical and chemical factors play crucial roles in regulating the interactions between predators and prey in the soil ecosystem (Bastida et al., 2021; Ding et al., 2022). For instance, pH, nutrient availability, and organic matter content have been found to be important drivers of protistan-fungal interactions in the soil (Zhou et al., 2021; Guo et al., 2022). The pH of the soil can affect both the growth and activity of protists and fungi, thereby influencing the strength and direction of their interactions (Örmälä-Odegrip et al., 2015; Rocca et al., 2022). Nutrient availability, particularly the availability of nitrogen (N) and phosphorus (P), has been found to be a key determinant of protistan-fungal interactions, with high N:P ratios favoring fungal growth and low ratios favoring protistan growth (Bonkowski, 2004; Gellner and McCann, 2016). In addition, organic matter content has been shown to influence the diversity and composition of soil microbial communities, which can in turn affect the interactions between predators and prey (Wall et al., 2015; Martin et al., 2023). Our results provide further evidence that these factors also influence the connectivity of bipartite networks (Table 1), which in turn can affect the structure and function of the soil ecosystem. For example, in healthy protistan-fungal networks, connectivity was significantly influenced by TK, AP, and AK. These findings suggest that increasing soil nutrient availability could promote the growth and activity of both protists and fungi, potentially leading to a more efficient transfer of energy and nutrients within the soil food web. In contrast, in infected soil, connectivity was significantly influenced by TOC, AK, and NH_4_^+^-N. This suggests that managing soil carbon and nitrogen availability could be particularly important for maintaining the stability and function of protistan-fungal networks in degraded soils. Overall, these findings highlight the complex nature of the interactions between predators and prey in the soil, and the importance of understanding the roles of soil physical and chemical factors in regulating these interactions. By identifying the specific soil variables that influence these interactions, we can develop more effective soil management practices that promote a healthy and diverse soil ecosystem.

Collectively, our study advances the understanding of the intricate interactions within soil microbial communities and their responses to the invasion of pathogenic fungi like *F. solani*. By elucidating the roles of different microbial groups, we provide insights into the dynamics of tobacco root rot disease. This knowledge holds significant implications for disease management strategies. The identification of potential key players within the microbial ecosystem, including both beneficial and pathogenic species, opens avenues for targeted interventions. Leveraging the biotic regulatory role of predatory protists and the intricate microbial interactions revealed in our study could lead to innovative approaches for controlling root rot outbreaks. Furthermore, understanding how environmental factors influence these interactions underscores the importance of maintaining optimal soil conditions to mitigate disease incidence. Overall, our findings offer practical insights that may contribute to the development of sustainable and effective strategies for managing root rot disease in tobacco cultivation.

## 5. Conclusion

Overall, this study demonstrates the complex interplay between soil physicochemical properties, microbial diversity, and plant health. The results suggest that the presence of pathogenic fungi can significantly alter the soil microbial community and increase soil nutrient levels. Additionally, the study highlights the importance of considering multiple factors, including pH and soil nutrients, in understanding the interactions between different microbial groups. The findings have implications for the development of sustainable agricultural practices that can promote soil health and prevent disease outbreaks in crops. Further research is needed to investigate the specific mechanisms underlying these complex microbial interactions and their effects on plant health.

## Data availability statement

The datasets presented in this study can be found in online repositories. The names of the repository/repositories and accession number(s) can be found in the article/Supplementary material.

## Author contributions

PL, TX, QH, YZ, and WM designed the experiments. TX, YY, ZW, XD, BW, and WL took samples and performed all data measurement. SG and PL contributed to the data analysis and wrote the paper. All authors contributed to the article and approved the submitted version.

## Funding

This research was supported by the Foundations for Tobacco Science of Wenshan Tobacco Company of Yunnan Province (2021530000241033) and Zhuzhou Tobacco Company of Hunan Province (22-004) of China.

## Conflict of interest

PL was employed by the Wenshan Tobacco Company of Yunnan Province.

The remaining authors declare that the research was conducted in the absence of any commercial or financial relationships that could be construed as a potential conflict of interest.

## Publisher’s note

All claims expressed in this article are solely those of the authors and do not necessarily represent those of their affiliated organizations, or those of the publisher, the editors and the reviewers. Any product that may be evaluated in this article, or claim that may be made by its manufacturer, is not guaranteed or endorsed by the publisher.

## Footnotes

^1^ http://mem.rcees.ac.cn:8080
