# Supplementary material for: Soil microbiota plays a key regulatory role in the outbreak of tobacco root rot
Source: Front Microbiol. 2023 Sep 15;14:1214167. doi: 10.3389/fmicb.2023.1214167 (PMC10540700; doi:10.3389/fmicb.2023.1214167)

**Supplementary Material**

**Soil microbiota plays a key regulatory role in the outbreak of tobacco root rot**

Pengfei Li ^1，†^, Songsong Gu ^3，†^, Yanmei Zhu ^1*^, Tianyang Xu ^1^, Yishuai Yang ^2^, Zhengqiang Wang ^2^, Xiangdong Deng ^2^, Bin Wang ^1^, Wei Li ^1^, Wenqiang Mei ^1，*^, Qiulong Hu ^2，*^,

*^1^ Wenshan Tobacco Company of Yunnan Province, Wenshan, Yunnan, China*

*^2^ Hunan Agricultural University, Changsha, Hunan, China*

*^3^Key Laboratory for Environmental Biotechnology, Research Center for Eco-Environmental Sciences, Chinese Academy of Sciences (CAS), Beijing, China.*

†These authors contributed equally to this work

* Correspondence author

^*^**Co-author for correspondence:**

Wenqiang Mei, Email: [12958297@qq.com](mailto:12958297@qq.com);

Qiulong Hu, Email: huqiulongnet@126.com

**Detailed information of the Supplementary Material**

The number of Tables: 5

The number of Figures: 7

Table S1. The dissimilarity test of microbial community structure between health and infected samples based on Bray-Curtis distance. The significant levels were described as 0.001***, 0.01**, and 0.05*. same as below.

Table S2. Mantel test between microbial communities and environmental factors. Environmental factors significantly correlated with the microbial community at the levels of 0.0001***, 0.01**, and 0.05* are in bold. Same as below.

Table S3. Empirical and random network properties of the bacterial, fungal and protistan communities. HS, the heathy soil; IS, the infected soil.

Table S4. Keystone species’ properties of all networks.

Table S5. Topological properties of intra-domain networks. 16S-ITS, the inter-domain network of prokaryotic-fungal community; 18S-ITS, the inter-domain network of protistan-fungal community.

Table S1.

| Methods | MRPP | | ANOSIM | | PERMANOVA | |
| --- | --- | --- | --- | --- | --- | --- |
|  | Delta | *P* | R | *P* | F | *P* |
| Bacteria | 0.4899 | 0.006****** | 0.2413 | 0.009****** | 2.9363 | 0.005****** |
| Fungi | 0.8497 | 0.003****** | 0.2473 | 0.008****** | 1.7453 | 0.003****** |
| Protist | 0.4914 | 0.017* | 0.2065 | 0.009** | 2.8355 | 0.015* |

Table S2.

| Groups | Bacteria | | Fungi | | Protist | |
| --- | --- | --- | --- | --- | --- | --- |
| env | r | *P* | r | *P* | r | *P* |
| pH | 0.2667 | **0.005**** | 0.2984 | **0.002**** | 0.1438 | **0.034*** |
| TOC | -0.0291 | 0.525 | 0.2382 | **0.033*** | -0.2329 | 0.969 |
| TN | 0.0327 | 0.336 | -0.0323 | 0.576 | -0.0537 | 0.598 |
| TP | 0.0401 | 0.332 | -0.1075 | 0.785 | 0.0155 | 0.412 |
| TK | 0.1413 | 0.151 | 0.1743 | 0.06 | 0.0044 | 0.429 |
| AP | 0.1618 | 0.12 | 0.0789 | 0.263 | 0.0982 | 0.212 |
| AK | 0.0854 | 0.225 | 0.1274 | 0.116 | 0.0045 | 0.414 |
| NO_3_^-^-N | 0.1187 | 0.094 | 0.1837 | **0.018*** | 0.1538 | **0.048*** |
| NH_4_^+^-N | 0.1111 | 0.128 | 0.1874 | **0.029*** | -0.0858 | 0.776 |

Table S3.

|  | 16S | | ITS | | 18S | |
| --- | --- | --- | --- | --- | --- | --- |
| Treatments | HS | IS | HS | IS | HS | IS |
| cutoff | 0.95 | 0.95 | 0.89 | 0.89 | 0.89 | 0.89 |
| Total nodes | 1286 | 1130 | 120 | 112 | 307 | 188 |
| Total links | 5993 | 1379 | 166 | 144 | 478 | 309 |
| R square of power-law | 0.971 | 0.981 | 0.827 | 0.902 | 0.886 | 0.860 |
| Average degree (avgK) | 9.320 | 2.441 | 2.767 | 2.571 | 3.114 | 3.287 |
| Average clustering coefficient (avgCC) | 0.371 | 0.196 | 0.179 | 0.065 | 0.287 | 0.309 |
| Average path distance (GD) | 6.667 | 12.582 | 5.646 | 4.935 | 8.542 | 8.104 |
| Transitivity (Trans) | 0.519 | 0.367 | 0.304 | 0.060 | 0.373 | 0.436 |
| Keystone species number | 29 | 8 | 5 | 10 | 4 | 1 |
| Modularity (fast_greedy) | 0.681 | 0.901 | 0.721 | 0.685 | 0.826 | 0.783 |
| Average clustering coefficient (avgCC) | 0.013 +/- 0.002 | 0.001 +/- 0.001 | 0.007 +/- 0.007 | 0.002 +/- 0.005 | 0.003 +/- 0.003 | 0.011 +/- 0.007 |
| Average path distance (GD) | 3.431 +/- 0.014 | 6.629 +/- 0.086 | 4.383 +/- 0.125 | 4.683 +/- 0.166 | 4.972 +/- 0.075 | 4.134 +/- 0.097 |
| Transitivity (Trans) | 0.042 +/- 0.001 | 0.005 +/- 0.002 | 0.038 +/- 0.014 | 0.022 +/- 0.013 | 0.016 +/- 0.005 | 0.041 +/- 0.011 |
| Modularity (fast_greedy) | 0.276 +/- 0.003 | 0.749 +/- 0.004 | 0.608 +/- 0.013 | 0.639 +/- 0.013 | 0.608 +/- 0.007 | 0.553 +/- 0.01 |

Table S4. Keystone species’ properties of all networks.

| Groups | Number | OTU | Domain | Phylum | Class | Order | Family | Genus | Species |
| --- | --- | --- | --- | --- | --- | --- | --- | --- | --- |
| 16S-HS | 1 | BOTU_1783 | Bacteria | Proteobacteria | Betaproteobacteria | Unclassified | Unclassified | Unclassified | Unclassified |
|  | 2 | BOTU_19083 | Bacteria | Verrucomicrobia | Subdivision3 | Unclassified | Unclassified | Subdivision3 genera incertae sedis | Unclassified |
|  | 3 | BOTU_2055 | Bacteria | Firmicutes | Bacilli | Bacillales | Planococcaceae | Planococcus | Unclassified |
|  | 4 | BOTU_514 | Bacteria | Proteobacteria | Alphaproteobacteria | Rhodospirillales | Acetobacteraceae | Unclassified | Unclassified |
|  | 5 | BOTU_600 | Bacteria | Proteobacteria | Betaproteobacteria | Burkholderiales | Oxalobacteraceae | Unclassified | Unclassified |
|  | 6 | BOTU_807 | Bacteria | Armatimonadetes | Chthonomonadetes | Chthonomonadales | Chthonomonadaceae | Chthonomonas/Armatimonadetes gp3 | Unclassified |
|  | 7 | BOTU_8425 | Bacteria | Candidatus Saccharibacteria | Unclassified | Unclassified | Unclassified | Saccharibacteria genera incertae sedis | Unclassified |
|  | 8 | BOTU_958 | Bacteria | Proteobacteria | Alphaproteobacteria | Rhodospirillales | Rhodospirillaceae | Unclassified | Unclassified |
|  | 9 | BOTU_9945 | Bacteria | Candidatus Saccharibacteria | Unclassified | Unclassified | Unclassified | Saccharibacteria genera incertae sedis | Unclassified |
|  | 10 | BOTU_10526 | Bacteria | Actinobacteria | Acidimicrobiia | Acidimicrobiales | Ilumatobacteraceae | Desertimonas | Unclassified |
|  | 11 | BOTU_1196 | Bacteria | Unclassified | Unclassified | Unclassified | Unclassified | Unclassified | Unclassified |
|  | 12 | BOTU_1205 | Bacteria | Proteobacteria | Betaproteobacteria | Burkholderiales | Comamonadaceae | Piscinibacter | Unclassified |
|  | 13 | BOTU_158 | Bacteria | Acidobacteria | Acidobacteria Gp1 | Unclassified | Unclassified | Gp1 | Unclassified |
|  | 14 | BOTU_179 | Bacteria | Proteobacteria | Betaproteobacteria | Unclassified | Unclassified | Unclassified | Unclassified |
|  | 15 | BOTU_180 | Bacteria | Proteobacteria | Betaproteobacteria | Rhodocyclales | Rhodocyclaceae | Unclassified | Unclassified |
|  | 16 | BOTU_2608 | Bacteria | Actinobacteria | Thermoleophilia | Gaiellales | Gaiellaceae | Gaiella | Unclassified |
|  | 17 | BOTU_34 | Bacteria | Bacteroidetes | Chitinophagia | Chitinophagales | Chitinophagaceae | Terrimonas | Unclassified |
|  | 18 | BOTU_342 | Bacteria | Acidobacteria | Acidobacteria Gp4 | Unclassified | Unclassified | Gp4 | Unclassified |
|  | 19 | BOTU_353 | Bacteria | Proteobacteria | Deltaproteobacteria | Unclassified | Unclassified | Unclassified | Unclassified |
|  | 20 | BOTU_4 | Bacteria | Gemmatimonadetes | Gemmatimonadetes | Gemmatimonadales | Gemmatimonadaceae | Gemmatirosa | Unclassified |
|  | 21 | BOTU_4463 | Bacteria | Gemmatimonadetes | Gemmatimonadetes | Gemmatimonadales | Gemmatimonadaceae | Gemmatirosa | Unclassified |
|  | 22 | BOTU_4725 | Bacteria | Acidobacteria | Acidobacteria Gp1 | Unclassified | Unclassified | Candidatus Koribacter | Unclassified |
|  | 23 | BOTU_48 | Bacteria | Bacteroidetes | Chitinophagia | Chitinophagales | Chitinophagaceae | Flavitalea | Unclassified |
|  | 24 | BOTU_4816 | Bacteria | Proteobacteria | Betaproteobacteria | Rhodocyclales | Rhodocyclaceae | Unclassified | Unclassified |
|  | 25 | BOTU_513 | Bacteria | Proteobacteria | Betaproteobacteria | Unclassified | Unclassified | Unclassified | Unclassified |
|  | 26 | BOTU_79 | Bacteria | Chloroflexi | Unclassified | Unclassified | Unclassified | Unclassified | Unclassified |
|  | 27 | BOTU_849 | Bacteria | Actinobacteria | Actinobacteria | Micromonosporales | Micromonosporaceae | Actinoplanes | Unclassified |
|  | 28 | BOTU_8510 | Bacteria | Proteobacteria | Betaproteobacteria | Unclassified | Unclassified | Unclassified | Unclassified |
|  | 29 | BOTU_9843 | Bacteria | Acidobacteria | Acidobacteria Gp3 | Unclassified | Unclassified | Candidatus Solibacter | Unclassified |
| 16S-IS | 1 | BOTU_1021 | Bacteria | Acidobacteria | Acidobacteria Gp1 | Unclassified | Unclassified | Unclassified | Unclassified |
|  | 2 | BOTU_124 | Bacteria | Acidobacteria | Acidobacteria Gp1 | Unclassified | Unclassified | Acidobacterium | Unclassified |
|  | 3 | BOTU_187 | Bacteria | Unclassified | Unclassified | Unclassified | Unclassified | Unclassified | Unclassified |
|  | 4 | BOTU_32 | Bacteria | Proteobacteria | Betaproteobacteria | Unclassified | Unclassified | Unclassified | Unclassified |
|  | 5 | BOTU_40 | Bacteria | Gemmatimonadetes | Gemmatimonadetes | Gemmatimonadales | Gemmatimonadaceae | Gemmatimonas | Unclassified |
|  | 6 | BOTU_434 | Bacteria | Actinobacteria | Thermoleophilia | Solirubrobacterales | Conexibacteraceae | Conexibacter | Unclassified |
|  | 7 | BOTU_4552 | Bacteria | Actinobacteria | Actinobacteria | Propionibacteriales | Propionibacteriaceae | Microlunatus | Unclassified |
|  | 8 | BOTU_5856 | Bacteria | Proteobacteria | Gammaproteobacteria | Nevskiales | Unclassified | Unclassified | Unclassified |
| ITS-HS | 1 | FOTU_543 | Fungi | Unclassified | Unclassified | Unclassified | Unclassified | Unclassified | Unclassified |
|  | 2 | FOTU_7556 | Fungi | Ascomycota | Pezizomycotina Incertae sedis | Pezizomycotina Incertae sedis | Pezizomycotina Incertae sedis | Ochroconis | Ochroconis constricta |
|  | 3 | FOTU_790 | Fungi | Ascomycota | Eurotiomycetes | Eurotiales | Trichocomaceae | Eupenicillium | Unclassified |
|  | 4 | FOTU_954 | Fungi | Ascomycota | Dothideomycetes | Dothideomycetes Incertae sedis | Myxotrichaceae | Gymnostellatospora | Gymnostellatospora japonica |
|  | 5 | FOTU_90 | Fungi | Unclassified | Unclassified | Unclassified | Unclassified | Unclassified | Unclassified |
| ITS-IS | 1 | FOTU_1029 | Fungi | Unclassified | Unclassified | Unclassified | Unclassified | Unclassified | Unclassified |
|  | 2 | FOTU_2043 | Fungi | Chytridiomycota | Chytridiomycetes | Spizellomycetales | Spizellomycetaceae | Spizellomyces | Unclassified |
|  | 3 | FOTU_2570 | Fungi | Basidiomycota | Agaricomycetes | Agaricales | Unclassified | Unclassified | Unclassified |
|  | 4 | FOTU_2755 | Fungi | Ascomycota | Sordariomycetes | Hypocreales | Hypocreales Incertae sedis | Unclassified | Unclassified |
|  | 5 | FOTU_584 | Fungi | Unclassified | Unclassified | Unclassified | Unclassified | Unclassified | Unclassified |
|  | 6 | FOTU_595 | Fungi | Unclassified | Unclassified | Unclassified | Unclassified | Unclassified | Unclassified |
|  | 7 | FOTU_10426 | Fungi | Ascomycota | Sordariomycetes | Hypocreales | Nectriaceae | Fusarium | Unclassified |
|  | 8 | FOTU_248 | Fungi | Basidiomycota | Unclassified | Unclassified | Unclassified | Unclassified | Unclassified |
|  | 9 | FOTU_4053 | Fungi | Unclassified | Unclassified | Unclassified | Unclassified | Unclassified | Unclassified |
|  | 10 | FOTU_5987 | Fungi | Basidiomycota | Agaricomycetes | Thelephorales | Thelephoraceae | Tomentella | Unclassified |
| 18S-HS | 1 | POTU_1399 | Rhizaria | Cercozoa | Filosa-Granofilosea | Filosa-Granofilosea X | Novel-Gran-5 | Novel-Gran-5 X | Novel-Gran-5 X sp. |
|  | 2 | POTU_105 | Alveolata | Dinoflagellata | Dinophyceae | Unclassified | Unclassified | Unclassified | Unclassified |
|  | 3 | POTU_25 | Alveolata | Ciliophora | Spirotrichea | Hypotrichia | Oxytrichidae | Oxytrichidae X | Oxytrichidae X sp. |
|  | 4 | POTU_46 | Opisthokonta | Metazoa | Tardigrada | Tardigrada X | Tardigrada XX | Tardigrada XXX | Tardigrada XXX sp. |
| 18S-IS | 1 | POTU_109 | Opisthokonta | Fungi | Ascomycota | Pezizomycotina | Sordariomycetes | Neurospora | Neurospora sitophila |

Table S5.

|  | 16S-ITS | | 18S-ITS | |
| --- | --- | --- | --- | --- |
|  | HS | IS | HS | IS |
| Cutoff | 0.8 | 0.8 | 0.7 | 0.7 |
| R square of power-law | 0.990 | 0.997 | 0.941 | 0.972 |
| number.of.species. Fungi | 96 | 72 | 131 | 127 |
| number.of.species. Bacteria or Protist | 714 | 522 | 344 | 188 |
| connectance | 0.019 | 0.021 | 0.029 | 0.030 |
| web asymmetry | -0.763 | -0.758 | -0.448 | -0.194 |
| links per species | 1.572 | 1.357 | 2.716 | 2.263 |
| number of compartments | 18 | 14 | 1 | 9 |
| cluster coefficient | 0.008 | 0.011 | 0.029 | 0.027 |
| nestedness | 1.421 | 1.930 | 6.251 | 4.385 |
| weighted nestedness | 0.485 | 0.457 | 0.182 | 0.225 |
| specialisation asymmetry | 0.365 | 0.348 | 0.292 | 0.168 |
| linkage density | 22.053 | 16.759 | 9.353 | 8.425 |
| weighted connectance | 0.027 | 0.028 | 0.020 | 0.027 |
| Shannon diversity | 7.149 | 6.692 | 7.162 | 6.569 |
| interaction evenness | 0.642 | 0.635 | 0.668 | 0.652 |
| mean.number.of.shared.partners.HL | 0.213 | 0.176 | 0.358 | 0.293 |
| mean.number.of.shared.partners.LL | 0.101 | 0.090 | 0.131 | 0.168 |
| robustness.HL | 0.594 | 0.560 | 0.726 | 0.703 |
| robustness.LL | 0.806 | 0.788 | 0.860 | 0.762 |
| functional.complementarity.HL | 333.492 | 239.198 | 442.542 | 298.127 |
| functional.complementarity.LL | 349.702 | 237.495 | 587.086 | 323.344 |
| partner.diversity.HL | 1.805 | 1.726 | 2.056 | 1.356 |
| partner.diversity.LL | 0.423 | 0.307 | 1.081 | 0.982 |
| Modularity | 0.735 | 0.746 | 0.535 | 0.589 |

Figure S1. The soil properties of healthy and infected samples, including pH, soil total organic carbon (TOC), soil organic matter (SOM), total nitrogen (TN), total phosphorus (TP), total potassium (TK), nitrate nitrogen (NO_3_^-^-N), ammonia nitrogen (NH_4_^+^-N), available phosphorus (AP), and available potassium (AK). HS, the healthy soil; IS, the infected soil. The significant levels were described as 0.001***, 0.01**, and 0.05*. Same as below.

Figure S2. The alpha diversity of microbial communities. (a) the prokaryotic community. (b) the fungal community. (c) the protistan community. HS, the healthy soil; IS, the infected soil.

Figure S3. The NMDS analysis based on Bray-curtis distance of microbial communities. (a) the prokaryotic community. (b) the fungal community. (c) the protistan community. HS, the healthy soil; IS, the infected soil.

Figure S4. The composition analysis at phylum level of microbial communities. (a) the prokaryotic community. (b) the fungal community. (c) the protistan community. HS, the healthy soil; IS, the infected soil.

Figure S5. the difference analysis based in response ratio and the composition analysis at species level of fungal communities. (a) the response ratio results of domain species (relative abundance >= 1%). (b) the composition distribution of domain fungal species. HS, the healthy soil; IS, the infected soil.

Figure S6. the variance partitioning analysis (VPA) between microbial community and environmental factors. (a) the results of prokaryotic community. (b) the results of fungal community. (c) the results of protistan community. Group 1 including pH, while group 2 including TOC, SOM, TN, TP, total potassium (TK), NO_3_^-^-N, NH_4_^+^-N, AP, and AK.

Figure S7. Sub-network of interactions between pathogens (red) and other fungal species in the root endophytic community of infected tobacco plant. Each node represents a species. Blue links represent positive correlations between nodes, and red links represent negatively correlated interaction relationship. (a) the sub-network of healthy samples. (b) the sub-network of infected samples.

Figure S1.


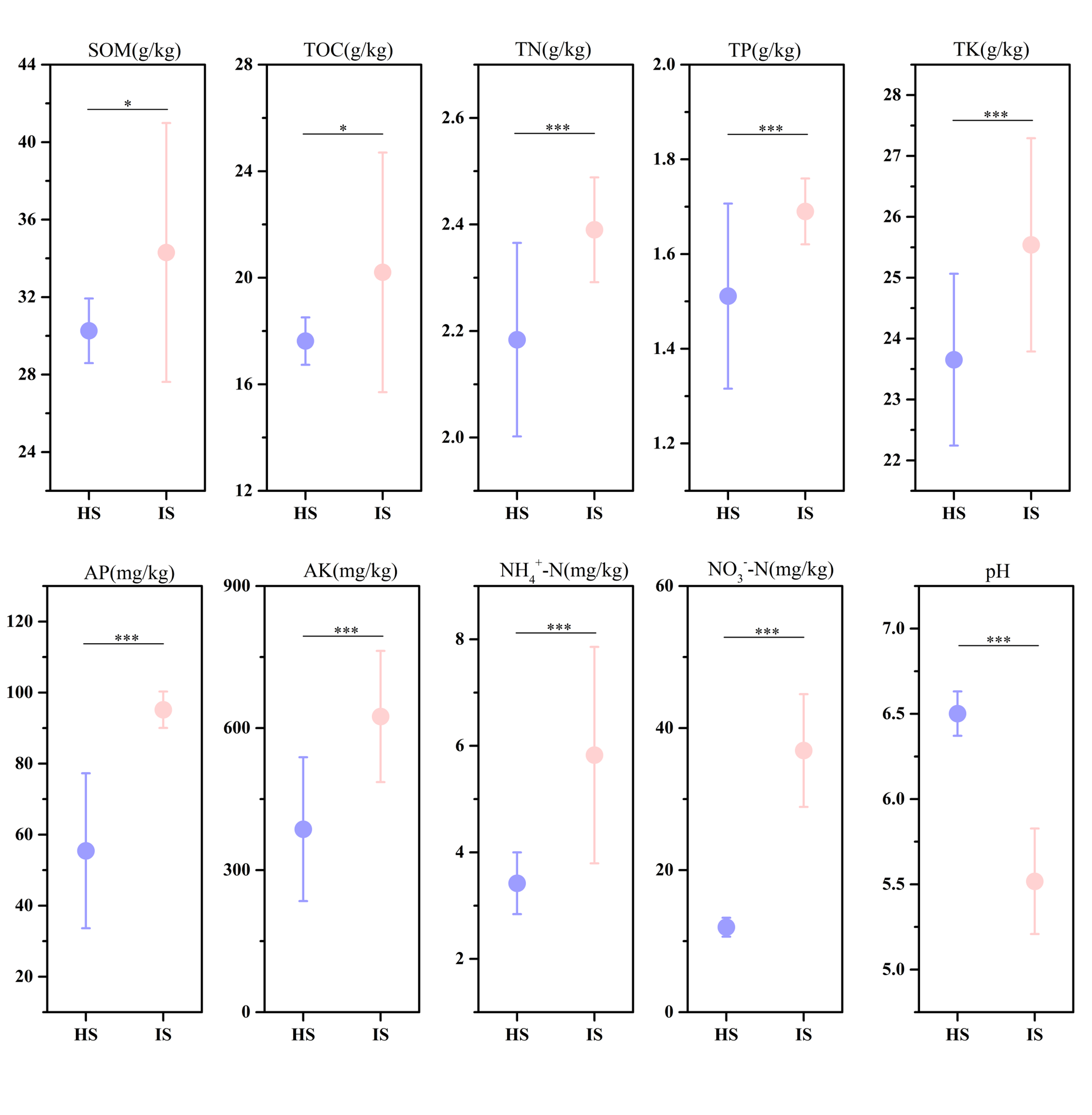


Figure S2.


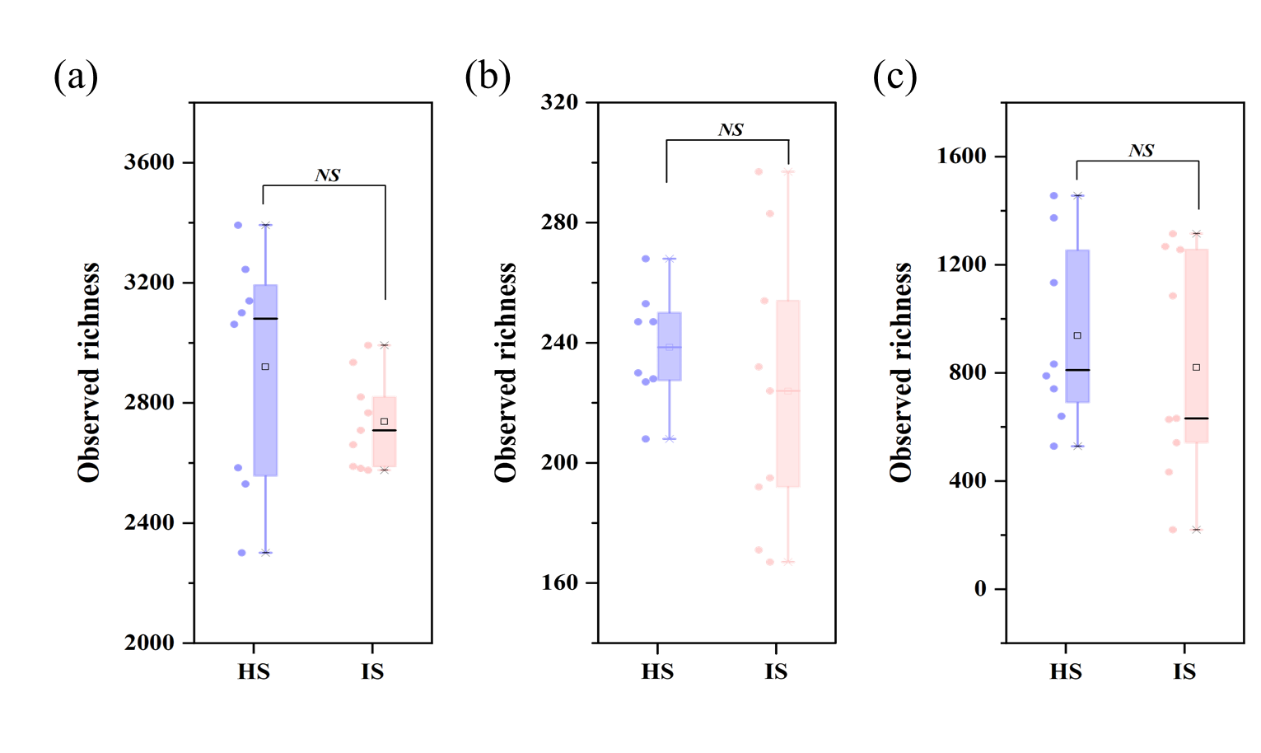


Figure S3.


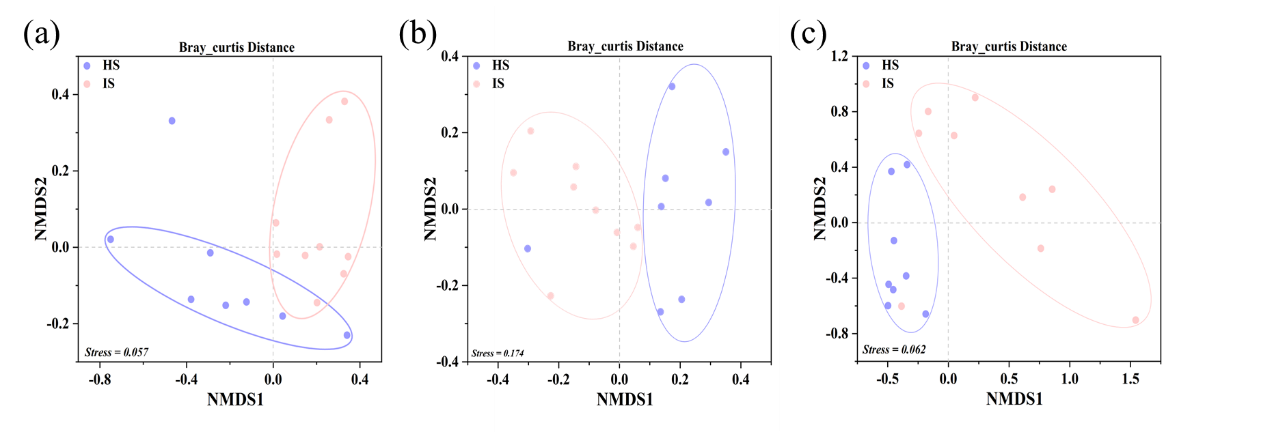


Figure S4.


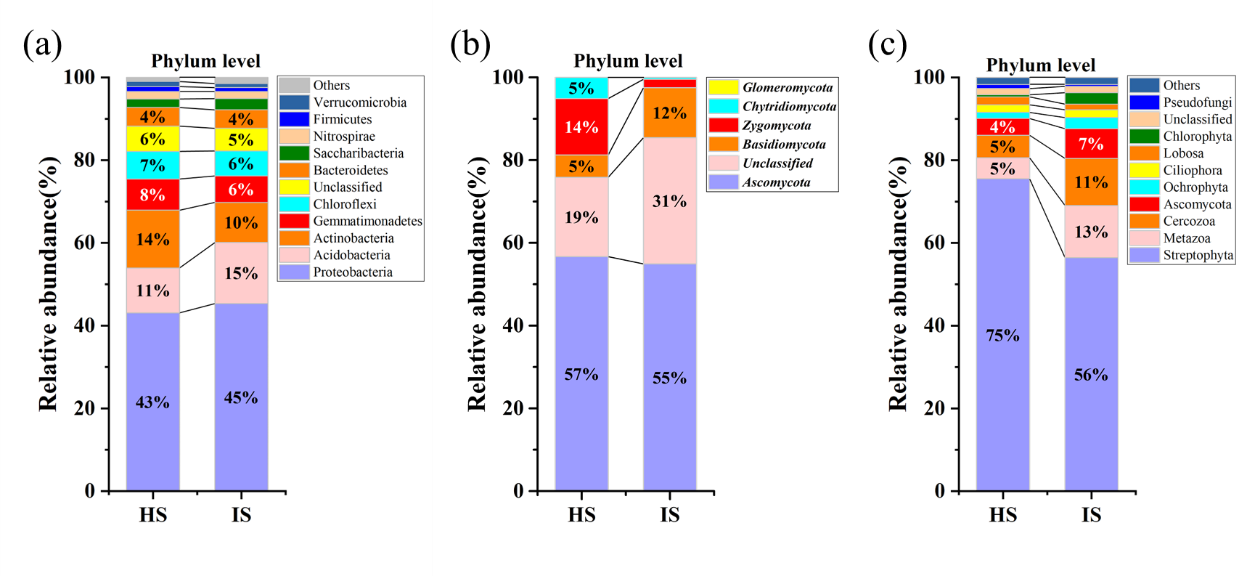


Figure S5.


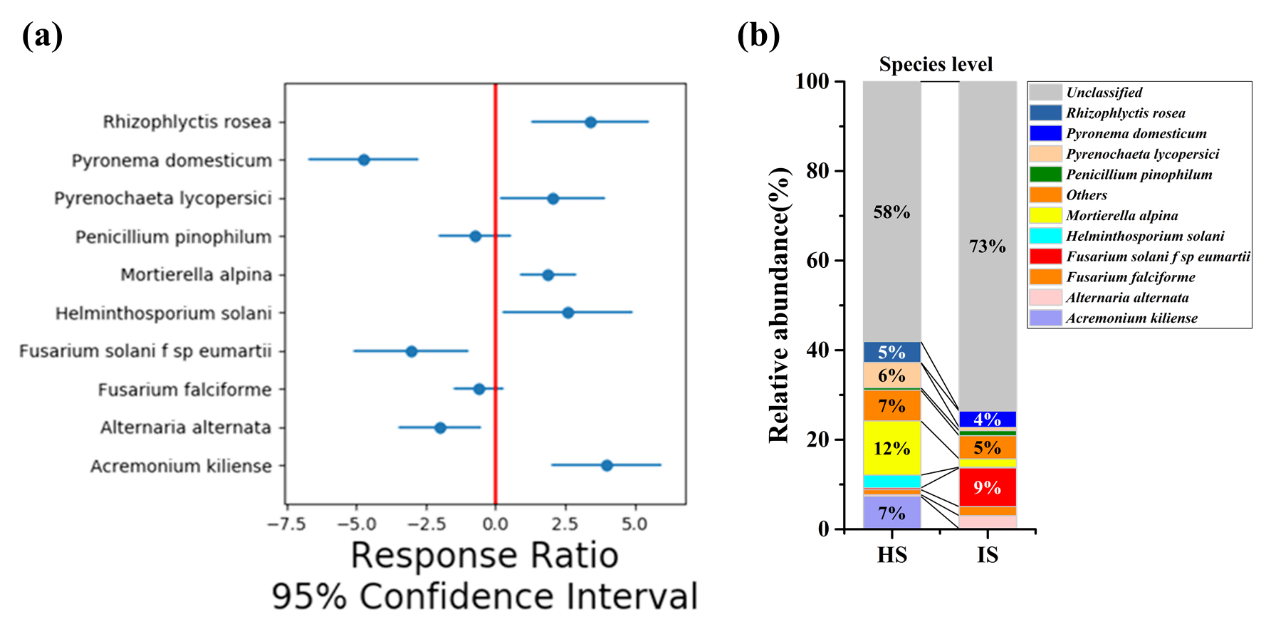


Figure S6.


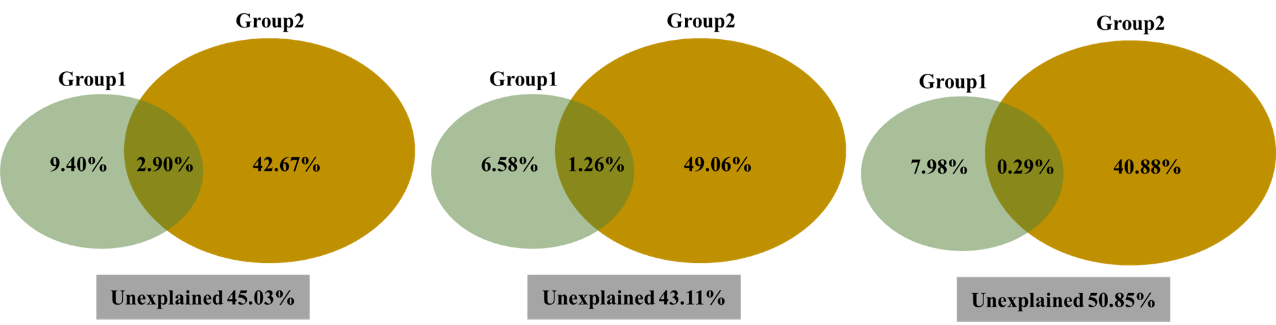


Figure S7.


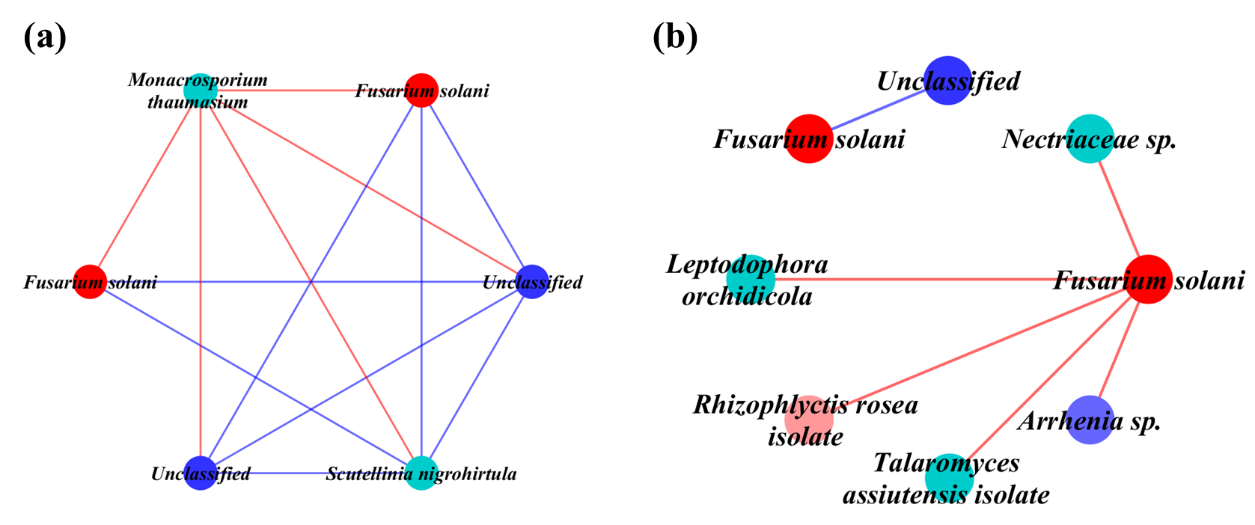

Supplement: Supplementary file 1 [file Data_Sheet_1.docx]
